# A facile and green method for synthesis of ZnFe_2_O_4_@CMC as a new magnetic nanophotocatalyst for ciprofloxacin removal from aqueous media

**DOI:** 10.1016/j.mex.2019.06.018

**Published:** 2019-06-27

**Authors:** Mohammad Malakootian, Alireza Nasiri, Ali Asadipour, Maryam Faraji, Elham Kargar

**Affiliations:** a Environmental Health Engineering Research Center, Kerman University of Medical Sciences, Kerman, Iran; b Department of Environmental Health, School of Public Health, Kerman University of Medical Sciences, Kerman, Iran; c Department of Medicinal Chemistry, Pharmaceutical Research Center, School of Pharmacy, Kerman University of Medical Sciences, Kerman, Iran

**Keywords:** Synthesis of ZnFe_2_O_4_@CMC as a new nanophotocatalyst by the hydrothermal method for photodegradation of ciprofloxacin, Antibiotic, ZnFe_2_O_4_@CMC, Hydrothermal method, Wastewater treatment, Langmuir-Hinshelwood model

## Abstract

This study aimed to synthesize a ZnFe_2_O_4_@carboxymethyl cellulose (CMC) nanophotocatalyst using a simple, facile, and green hydrothermal method for ciprofloxacin (CIP) removal from aqueous solutions. At first, ZnFe_2_O_4_@CMC was synthesized using the hydrothermal method, and then it was characterized. To assay the photocatalytic activity of ZnFe_2_O_4_@CMC, its ability was investigated in CIP and total organic carbon (TOC) removal by considering the effect of some variables such as initial CIP concentrations (5–30 mg/L), pH (3–11), nanophotocatalyst loading (0.1–0.5 g), and reaction time (20–120 min). The kinetic performance of the process was assessed by the *pseudo-*first order and Langmuir-Hinshelwood models. The concentration of CIP and TOC in the samples was determined using high performance liquid chromatography and a TOC analyzer, respectively. The influence of competitive compounds was examined on the efficiency of the nanophotocatalyst through its application for a real wastewater sample. Moreover, the reusability and chemical stability of ZnFe_2_O_4_@CMC were studied.

Advantages of this technique are as follows:

• ZnFe_2_O_4_@CMC as a high potent magnetically separable photocatalyst is designed by a green, simple, and fast hydrothermal method.
• ZnFe_2_O_4_@CMC displays significant photocatalytic activity in photocatalytic degradation processes for ciprofloxacin removal.
• ZnFe_2_O_4_@CMC exhibits good chemical stability and reusability.

## Graphical abstract

**Figure:**
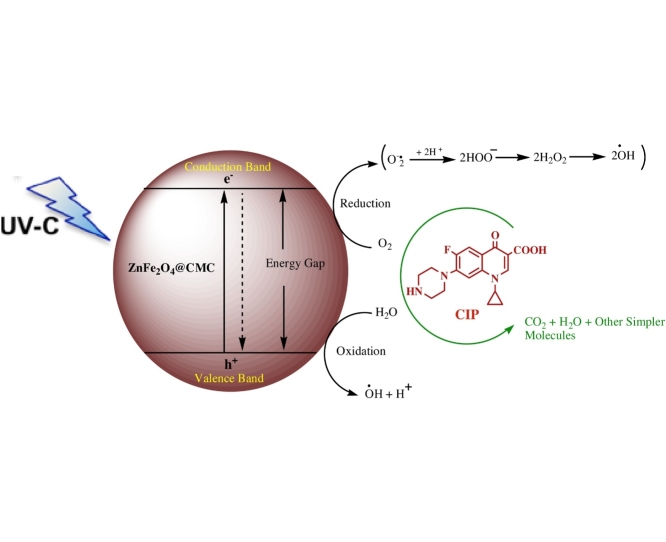

**Specifications Table**

**Table:** 

Subject Area:	*Environmental Science*
More specific subject area:	*Chemical engineering in environmental sciences*
Method name:	*Synthesis of ZnFe_2_O_4_@CMC as a new nanophotocatalyst by the hydrothermal method for photodegradation of ciprofloxacin*
Name and reference of original method:	*Malakootian M, Nasiri A, Asadipour A, & Kargar E. Facile and green synthesis of ZnFe_2_O_4_@CMC as a new magnetic nanophotocatalyst for ciprofloxacin degradation from aqueous media. Process Safety and Environmental Protection. (2019) In press.*
Resource availability:	*N/A*

## Method details

**Fig. 1:**
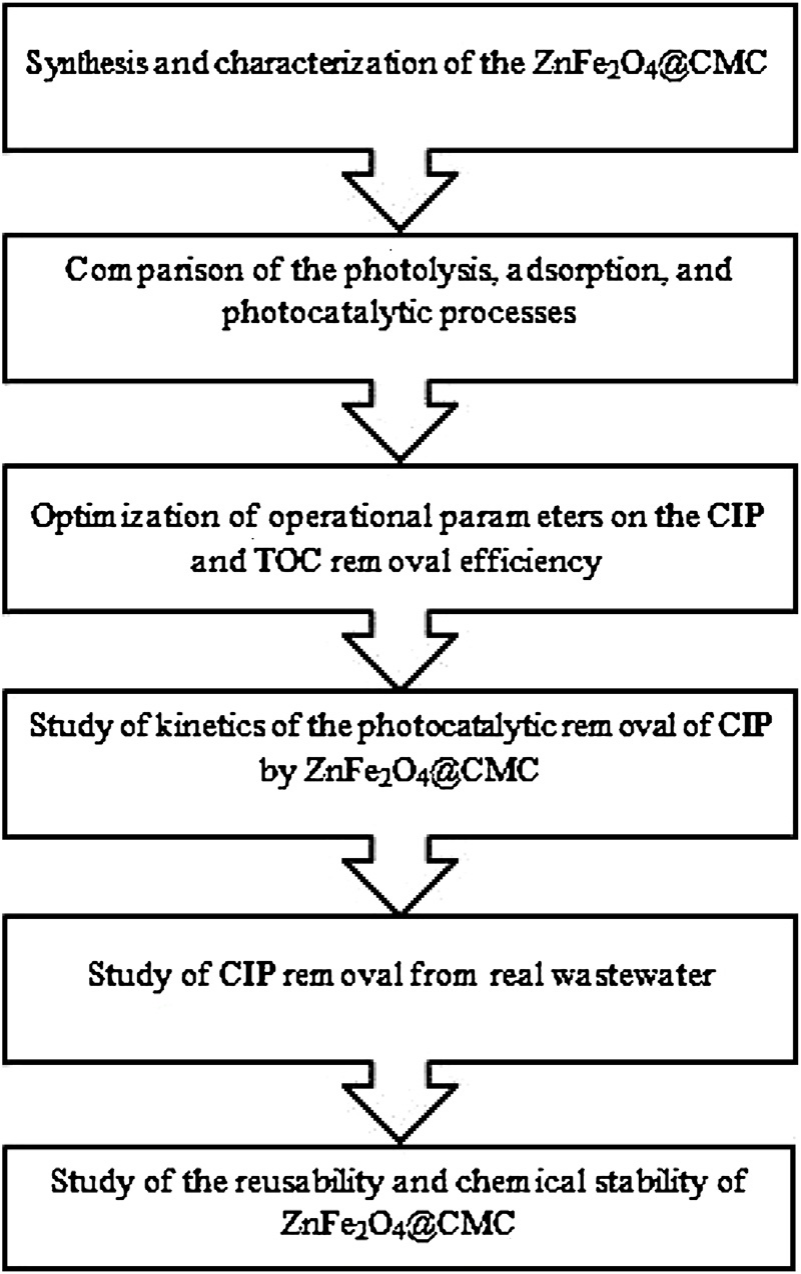
The flow diagram of the study stages.

The study stages were as follows: synthesis and characterization of ZnFe_2_O_4_@CMC; comparison of photolysis, adsorption, and photocatalytic processes; study of effects of operational parameters on ciprofloxacin (CIP) and total organic carbon (TOC) removal efficiency; study of kinetics of the photocatalytic removal of CIP by ZnFe_2_O_4_@CMC; study of CIP removal from real wastewater and study of the recovery, reusability and chemical stability of ZnFe_2_O_4_@CMC (Fig. 1). To the best of our knowledge, there has been no investigation on the synthesis of ZnFe_2_O_4_@CMC as a magnetic nanophotocatalyst. Moreover, carboxymethyl cellulose carbohydrate was used as a biopolymer to improve the structural and photocatalytic properties of a nanophotocatalyst for the first time in the current study. In this research, ZnFe_2_O_4_@CMC was synthesized using the hydrothermal method. In this method, no toxic solvent is used to synthesize magnetic nanobiocomposites. The procedure was carried out by using water as a solvent as complicated methods are not required for the preparation of ZnFe_2_O_4_@CMC. The photocatalyst was produced from available materials such as iron and zinc nitrate salts and CMC in alkaline conditions. Thus, this method can be defined as a facile and green method to synthesize ZnFe_2_O_4_@CMC.

## Chemicals

Ciprofloxacin (99%) was obtained from Temad Pharmaceutical Company (Tehran, Iran), and CMC was purchased from Sigma-Aldrich Company (USA). Other chemicals such as Fe(NO_3_)_3_·9H_2_O, Zn(NO_3_)_2_·6H_2_O, HCl, NaOH, acetonitrile, acetic acid, methanol, and ethanol were obtained from Merck Company (Germany). All the materials were in analytical grade and used without further purification. All the aqueous solutions were prepared using distilled water.

## Synthesis and characterization of ZnFe_2_O_4_@CMC

**Fig. 2:**
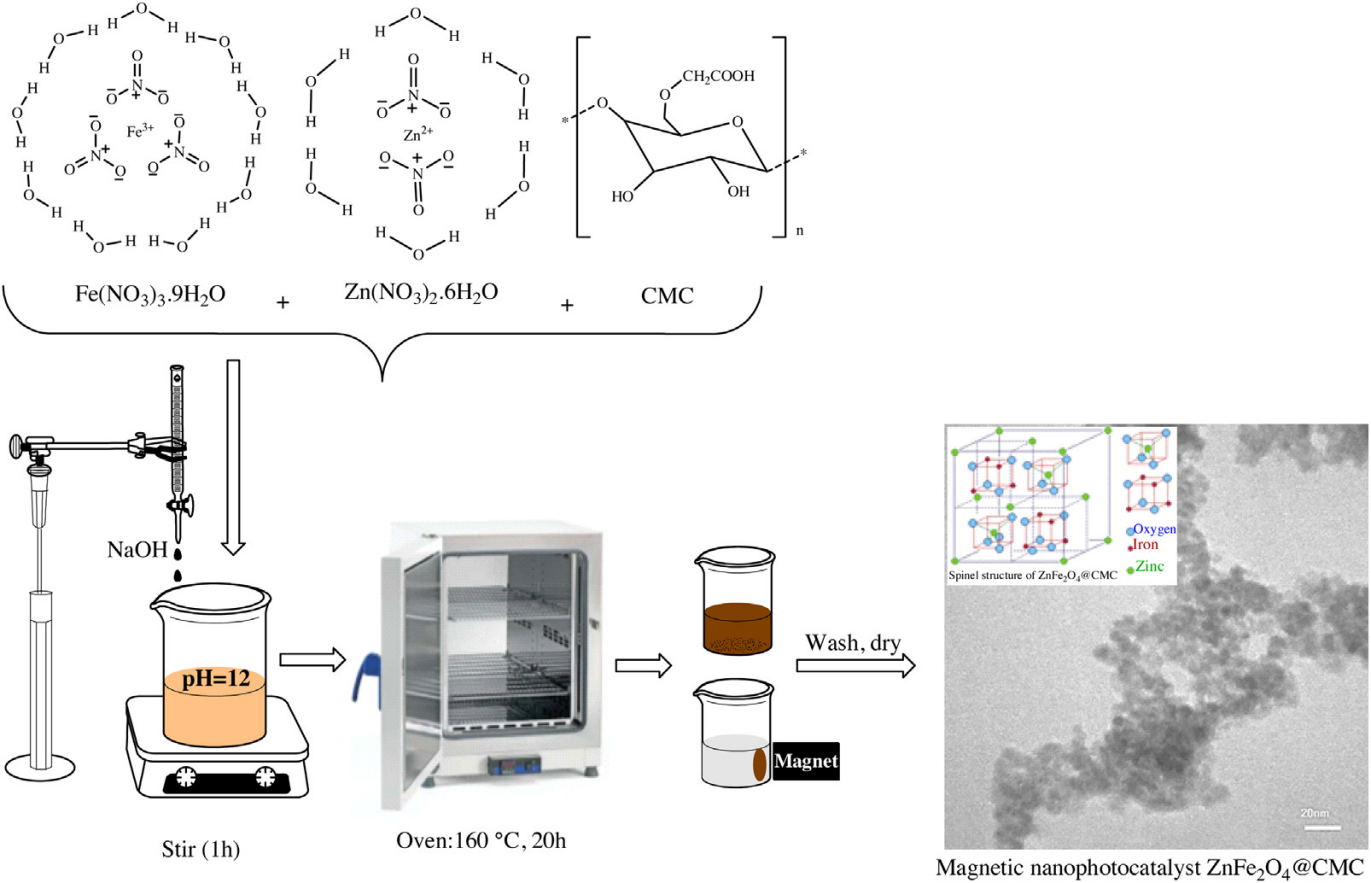
The schematic illustration of the synthesis of ZnFe_2_O_4_@CMC as a new magnetic nanophotocatalyst.

Synthesis of ZnFe_2_O_4_@CMC through using the hydrothermal method was described in detail in an article [1]. Briefly, Fe(NO_3_)_3_·9H_2_O (8.06 g) and Zn(NO_3_)_2_·6H_2_O (2.96 g) in a 2:1 ratio were dissolved in 100 mL deionized water. Then, 0.5 g of CMC was added to the solution and the mixture was vigorously stirred at room temperature. Subsequently, 6 g of NaOH was added to the mixture gradually within an hour to obtain a brown suspension with pH = 12. Afterwards, the obtained brown suspension was placed in an oven at 160 °C for 20 h. The resulting precipitate was washed several times with distilled water and ethanol and dried at 60 °C for 2 h (Fig. 2).

ZnFe_2_O_4_@CMC was characterized by X-ray powder diffraction (XRD) in the diffraction angle range 2θ = 20°–80° by an X’Pert PRO MPD P analytical using Ni-FILTERED Cu Kα radiation, field emission scanning electron microscope-energy dispersive spectroscopy (FESEM-EDS (MIRA3TESCANXMU), transmission electron microscopy (TEM, Philips CM30 unit operated at 150 kV), Fourier transform infrared spectroscopy (FT-IR; 6300 Brucker), vibrating sample magnetometer (VSM; LakeShore Cryotronics-7404), UV–vis diffuse reflectance spectra (UV-DRS; UV–vis spectrophotometer, Shimadzu, UV-2550), energy-dispersive X-ray spectroscopy (EDS) and mapping (MIRA3TESCAN-XMU). The BET surface areas were also evaluated based on N_2_ adsorption–desorption isotherms using a specific surface analyzer (BELSORP-mini II) at 120 °C [1].

## Comparison of the photolysis, adsorption, and photocatalytic processes

In studies on photocatalysis processes, comparison of the results of photolysis, adsorption and photocatalytic mechanisms is important [2]. Therefore, the mentioned processes were respectively differentiated in the experiments in presence of UV radiation, but without a catalyst to assay the photolysis process, and in dark condition to evaluate the adsorption process.

## Optimization of operational parameters on the CIP and TOC removal efficiency

The effects of operational parameters such as initial CIP concentrations (5, 10, 20, 30 mg/L), pH (3, 5, 7, 9, 11), nanophotocatalyst loading (0.1, 0.2, 0.3, 0.5 g), and reaction time (20, 40, 60, 80, 100, 120 min) were optimized in a batch borosilicate glass photoreactor (internal dimensions with length: 25 cm, width: 10 cm and height: 5 cm) equipped with three UV lamps (6 W, Philips), a mechanical stirrer and a cooling water chamber to keep temperature in a constant value.

The mineralization ability is a substantial parameter to assess photocatalytic properties of synthesized photocatalysts. TOC removal was investigated by photolysis, adsorption and photocatalytic processes. The photocatalytic process of UV/ZnFe_2_O_4_@CMC can effectively remove TOC 75% in the photocatalyst of 0.3 g at pH = 7, initial CIP concentration of 5 mg/L and irradiation time of 100 min (optimal condition).

**Fig. 3:**
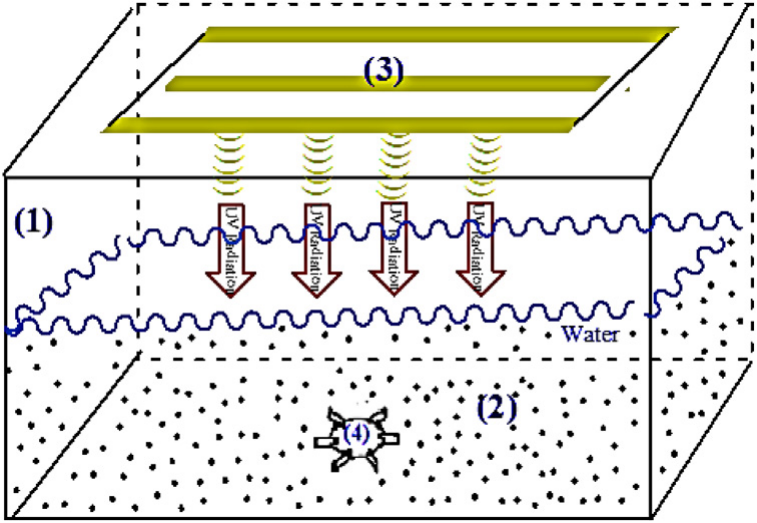
The photoreactor designed for the photocatalytic degradation of CIP; (1) the Plexiglas reactor, (2) the photocatalyst, (3) the UV-C lamp, and (4) the mechanical stirrer.

The photoreactor designed for the current study is shown in Fig. 3. ZnFe_2_O_4_@CMC was separated from the effluent by an external magnet and analyzed by HPLC. The samples were taken at the deﬁnite interval of times during the irradiation and, after the separation of ZnFe_2_O_4_@CMC by an external magnet, were analyzed by high performance liquid chromatography (HPLC- Waters E600, USA). Details of the HPLC analysis are provided in Table 1. Then, degradation efficiency was calculated by Eq. (1):

(1) Degradation efficiency % = 100 (C_0_ − C_t_)/C_0_

where C_t_ and C_0_ are the obtained concentration of the CIP solution at t and 0 min by HPLC, respectively [3]. Moreover, TOC was measured in the samples by the TOC analyzer (Shimadzu TOC‒VCSH).

**Table 1:** Details of the HPLC analysis.

Characteristic	Condition
Detector	UV absorbance at the wavelength of 272 nm
Column model	C18 column with 5-μm particles
Column characteristic	250 mm length and 4.6 mm internal diameter
Mobile phase	Water/methanol/acetonitrile/1% acetic acid (15/15/20/50, V/V)
Flow rate of mobile phase	0.5 mL/min
Volume of injection	60 μL

## Study of kinetics of the photocatalytic removal of CIP by ZnFe_2_O_4_@CMC

The kinetics of the photocatalytic removal of CIP by ZnFe_2_O_4_@CMC was studied by the *pseudo-*first order (Eq. (2)) and Langmuir-Hinshelwood (L-H) (Eq. (3)) kinetic models, as described in Table 2.

(2) Ln (C_t_/C_0_) = −K_obs_t

where C_0_, C_t_, and K_obs_ are the initial concentrations of CIP, CIP concentration at certain reaction times, and constant rate of the *pseudo-*first order reaction, respectively [4].

(3) $$\frac{1}{K_{obs}}=\frac{1}{K_{C}K_{L-H}}+\frac{C_{0}}{K_{C}}$$

where C_0_ is the initial CIP concentration, K_c_ is the constant rate of the superficial reaction (mg L^−1^ min^−1^), and K_L-H_ is the adsorption equilibrium constant of the L-H model (L mg^−1^) [4].

**Table 2:** *Pseudo-*first order and Langmuir-Hinshelwood (L-H) kinetic models.

Model	Formula	Parameters
*Pseudo-*first order	Ln (C_t_/C_0_) = −K_obs_t	C_0_ (mg/L): initial concentrations of CIP
		C_t_ (mg/L): CIP concentration at certain reaction times
		K_obs_ (min^−1^): constant rate of the *pseudo-*first order reaction
		t (min): reaction time
Langmuir-Hinshelwood	$\frac{1}{K_{\text{obs}\;}}=\frac{1}{K_{c}K_{L-H}}+\frac{C_{0}}{K_{c}}$	K_c_ (mg/L min): constant rate of the superficial reaction
		K_L-H_ (L/mg): adsorption equilibrium constant of the L-H model

## Study of CIP removal from real wastewater

It is important to be able to apply a nanophotocatalyst for treatment of real wastewaters in presence of competitive compounds. Thus, the efficiency of the photocatalytic process of UV/ZnFe_2_O_4_@CMC in CIP removal was determined in optimal conditions at the sewerage network at the campus of the Kerman University of Medical Sciences (CIP = 4.5 mg/L, COD = 415 mg/L, BOD = 247 mg/L).

## Study of the reusability and chemical stability of ZnFe_2_O_4_@CMC

Reusability is a main factor for practical applications of heterogeneous magnetic catalysts [5]. Therefore, reusability of the ZnFe_2_O_4_@CMC photocatalyst was investigated for the photocatalytic degradation of CIP in five runs. After each run, ZnFe_2_O_4_@CMC was separated by an external magnetic field, washed with alcohol/water, dried at 100 ^○^C and then reused. Finally, the removal efficiency of CIP in each run was compared with the other runs.

The chemical stability of ZnFe_2_O_4_@CMC was investigated after five recovery runs. Moreover, the XRD analysis of the ZnFe_2_O_4_@CMC photocatalyst was carried out after five runs. There was no change in the crystalline structure of ZnFe_2_O_4_@CMC. Moreover, the stability of the photocatalyst was examined by measuring the concentration of Fe and Zn ions after degradation in the solution by an atomic absorption spectrometer (AAS-PG Instruments, model PG 990-England) at the wavelengths of 248.3 nm and 213.9 nm, respectively, in the last run. These results indicated that this photocatalyst could be easily recovered and, after being reused for five runs, showed good chemical stability, which would promote its industrial applications in antibiotic degradation from pharmaceutical wastewaters [6].

## References

[1] Malakootian M., Nasiri A., Asadipour A., Kargar E. (2019). Facile and green synthesis of ZnFe_2_O_4_@CMC as a new magnetic nanophotocatalyst for ciprofloxacin degradation from aqueous media. Process. Saf. Environ. Prot..

[2] Malakootian M., Nasiri A., Amiri Gharaghani M. (2019). Photocatalytic degradation of ciprofloxacin antibiotic by TiO_2_ nanoparticles immobilized on a glass plate. Chem. Eng. Commun..

[3] Malakootian M., Olama N., Malakootian M., Nasiri A. (2018). Photocatalytic degradation of metronidazole from aquatic solution by TiO_2_-doped Fe^3+^ nano-photocatalyst. Int. J. Environ. Sci. Technol..

[4] Nasiri A., Tamaddon F., Mosslemin M.H., Amiri Gharaghani M., Asadipour A. (2019). Magnetic nano-biocomposite CuFe_2_O_4_@methylcellulose (MC) prepared as a new nano-photocatalyst for degradation of ciprofloxacin from aqueous solution. Environ. Health Eng. Manage. J..

[5] Nasiri A., Tamaddon F., Mosslemin M.H., Gharaghani M.A., Asadipour A. (2019). New magnetic nanobiocomposite CoFe_2_O_4_@methycellulose: facile synthesis, characterization, and photocatalytic degradation of metronidazole. J. Mater. Sci. Mater. Electron..

[6] Malakootian M., Yaseri M., Faraji M. (2019). Removal of antibiotics from aqueous solutions by nanoparticles: a systematic review and meta-analysis. Environ. Sci. Pollut. Res. - Int..

